# Calcium in Neuronal and Glial Response to Axotomy

**DOI:** 10.3390/ijms222413344

**Published:** 2021-12-12

**Authors:** Andrey Khaitin

**Affiliations:** Academy of Biology and Biotechnology, Southern Federal University, 344090 Rostov-on-Don, Russia; andrej.wojt@gmail.com

**Keywords:** axotomy, calcium, glia, neurotrauma

## Abstract

Neurotrauma assumes an instant or delayed disconnection of axons (axotomy), which affects not only neurons, but surrounding glia as well. Not only mechanically injured glia near the site of disconnection, especially transection, is subjected to the damage, but also glia that is remote from the lesion site. Glial cells, which surround the neuronal body, in turn, support neuron survival, so there is a mutual protection between neuron and glia. Calcium signaling is a central mediator of all post-axotomy events, both in neuron and glia, playing a critical role in their survival/regeneration or death/degeneration. The involvement of calcium in post-axotomy survival of the remote, mechanically intact glia is poorly studied. The purpose of this review is to sum up the calcium-involving mechanisms in responses of neurons and glial cells to axotomy to show their importance and to give some suggestions for future research of remote glia in this context.

## 1. Introduction

Traumatic injuries to the central nervous system (brain and spinal cord) affect young and middle age people causing premature death and disability [1,2]. These injuries, as well as peripheral nerve injuries because of wounds or surgeries, involve axotomy (AT), i.e., transection or disconnection of axons. AT is followed by either degeneration and death of neurons, or regeneration of axons and their reconnection with their targets. To fight the consequences of neurotraumas, it is necessary to stop the processes, leading to neuron death, as soon as possible. Unfortunately, to date, no sufficiently effective neuroprotectors have been discovered. A more profound study of molecular processes, induced by axon lesion, is required.

The neurons’ vulnerability to AT depends on a number of factors, such as localization, age, and distance. Neurons in the peripheral nervous system (PNS) usually survive axotomy and regenerate, while many neurons in the central nervous system (CNS) undergo degeneration and death after axotomy. This is related to neuronal factors, such as differences in gene expression in cell response to axotomy, and non-neuronal factors, such as immune proteins, which inhibit the regeneration, and the interaction of both types of factors [3,4]. In very young animals, axon damage results in retrograde degeneration and death both in the PNS and CNS [5]. Generally, the more remote the lesion is from the soma, the more resistant to axotomy is the neuron [6,7]. There are two main suggestions for possible mechanisms of how the information about the trauma is transmitted to a cell body: (a) a mechanism of double signals, where the distance to the lesion site is estimated based on the time delay between lesion-induced early and rapid ion fluxes and the later arrival of motor-dependent signal complexes; and (b) a mechanism involving continuous scanning and regulation of axonal transport in two directions [5,8].

Two forms of axotomy are now considered. “Primary” axotomy occurs when axons are broken apart or transected directly in the mechanical impact on nervous tissue. The physical transection of axons massively involves its microenvironment and creates a dramatic, immediate disturbance of ionic regulation. “Secondary” axotomy occurs after relatively minor lesions to axons, such as stretch injury or diffuse axonal injury in brain trauma, which trigger a cascade of events, ultimately resulting in cytoskeleton degradation and axonal rupture [9]. In the CNS, primary axotomy is more typical for spinal cord injury, while secondary axotomy is more typical for traumatic brain injury. There are very little data on secondary axotomy in the PNS.

Ca^2+^ is critically involved in a number of signal pathways, controlling cell homeostasis. It plays an important role in neurodegeneration [10,11], particularly, in the response of neurons to AT [2,8,9]. The elevation of cytosolic calcium concentration from 10^−4^–10^−3^ M can trigger processes of cell death, necrosis or apoptosis [12].

The injury-induced focal permeability leads to local Ca^2+^ influx with activation of cysteine proteases, calpain and caspases, which play an important role in resulting pathogenesis of traumatic axon injury via the proteolytic cleavage of cerebral spectrin, one of the components of subaxolemmal cytoskeleton. During this pathological process, a local calcium overload, together with calpain activation, also causes mitochondrial damage, resulting in the release of cytochrome c and caspase activation. Then the activated calpain and caspases are involved in the degradation of the local axonal cytoskeleton [13].

Ca^2+^ signaling is very important in the glial environment of neurons, which plays a substantial role in the survival and regeneration of neurons after injury [14]. The injury-induced Ca^2+^-related transcription factors can serve as useful biomarkers of pathological processes in reactive glia [15]. It has been shown that damage to glia may suppress neuronal functions and induce neuron loss [16]. Moreover, damage to nerves induces the death of surrounding glial cells [17]. What is important, damage to nerves not only causes death of glial cells, immediately adjacent to the affected area (or damaged collaterally in mechanical way), but also to glial cells, remote from the damage site (remote glial cells, further RGC). Ca^2+^ is one of the intermediaries between cellular–molecular events in the damage site and in RGC. The exact role of Ca^2+^, including extracellular calcium, the different mechanisms of cytosolic Ca^2+^ regulation (Ca^2+^ channels and pumps), and Ca^2+^-activated proteins in the survival and death of RGC are still unclear.

## 2. Calcium Dynamics in Neurons and Glia after Axotomy

In the experiments of Ziv and Spira on isolated Aplysia neurons [18], the spatiotemporal dynamics of intra-axonal calcium levels from the transection site was studied. The dynamics of Ca^2+^ were similar in both cut ends and went through the following stages: axolemmal disruption and up to a 30-fold Ca^2+^ elevation along the whole axon; and the slower process propagation of Ca^2+^ elevation front propagation with 11–16 pm/sec speed from the transection site towards intact end. After the sealing of the axonal lumen, the Ca^2+^ level recovered to initial values for 7–10 min, going from intact ends to lesion sites. In the absence of Ca^2+^ in the medium, axon transection does not result in Ca^2+^ elevation and lumen resealing. After the returning of normal levels of Ca^2+^ in the medium, Ca^2+^ is increased near the transected ends and the lumen is sealed.

The AT-induced Ca^2+^ elevation is mainly provided by the influx of Ca^2+^ through voltage-gated Ca^2+^ channels, inversion of Na^+^–Ca^2+^ exchanger, and the lumen. However, the spatiotemporal dynamics of Ca^2+^ after axon transection does not correspond to just diffusion, suggesting that Ca^2+^ gradients are created and restricted through some other mechanisms, making it possible for the neuron to survive the injury and ultimately recover. Ca^2+^ ions mediate early events in axo-somal communication (retrograde signaling) after nerve damage. Rishal and Fainzilber [8] consider in detail the mechanism of the retrograde Ca^2+^ front that is formed after axon transection, involving the possible reinforcement via the release of Ca^2+^ from intracellular storages, such as endoplasmic reticulum. The resulting Ca^2+^ waves propagate along the axon and serve as the initial damage signal for soma. In our experiments on crayfish stretch receptor neurons, axotomy induced the elevation in cytosolic calcium levels in soma and near-soma axoplasm within minutes [19].

The increase in cytosolic calcium concentration also induces calcium-activated chlorine currents [20]. In crayfish motor neurons, a high increase in the expression of chlorine channels was observed [21].

As a result of focal brain trauma, extracellular calcium decreases to 0.1 mM [22]. [Ca^2+^]_o_ elevation increases pH_i_ and decreases [Na^+^]_i_, and vice versa [23].

AT promotes the activity of the plasmatic membrane Ca^2+^ ATPase (PMCA), which regulates intracellular Ca^2+^ concentration by taking calcium out from the cell [24]. In addition, AT promotes the expression of PMCA in dorsal root ganglia [25].

In mammal glial cells, ER lumen is one of the main sources of signal transduction Ca^2+^. Upon depletion, the lumen is filled with Ca^2+^ ions from the extracellular space via the SOCE mechanism (store-operated calcium entrance) [26].

Astrocytes exchange signals via ATP. IP3 molecule messengers diffuse between astrocytes through gap junctions. IP3 activates Ca^2+^ channels in cell organelles, which results in the release of Ca^2+^ to cytosol. This Ca^2+^ can additionally promote IP3 production and cause ATP release through membrane channels, formed by pannexins and innexins [14]. This eventually results in a Ca^2+^ wave, propagating from cell to cell. In addition, the wave can be mediated by the release of ATP to the extracellular medium and following purinergic receptor activation. The NFAT transcription factor links Ca^2+^ signaling with reactive transcriptional changes. Blockade of astrocytic calcineurin/NFAT signaling helps to normalize hippocampal synaptic function and plasticity in a rat model of traumatic brain injury [15].

Satellite glial cells are small cells surrounding neurons in sensory, sympathetic, and parasympathetic ganglia. These cells are involved in the chemical environment regulation, in particular, buffering it with the help of K^+^ and Ca^2+^ channels [27]. Similar to astrocytes, they are connected with each other via gap junctions and respond to ATP signals, increasing intracellular Ca^2+^. They are highly inflammation-sensitive and contribute to pathological conditions, such as chronic pain.

Although neurons and satellite glia are not strongly connected or coupled, their close location provides favorable conditions for effective communication. Bidirectional Ca^2+^ signaling between satellite glial cells and neurons, involving both gap junctions and ATP release, is detected [28,29]. However, it is still unknown how the activation of satellite glia after axonal damage is associated with neuroglial communication.

Unlike glial cells, located in the lesion site or in immediate proximity, which are directly damaged in axotomy, the sensibly distant glial cells can be susceptible to the injury only indirectly. The question of what is happening to this glia, in particular, with glial envelope around soma and proximal axon area of the neuron, is little studied. Very little is particularly known about how Ca^2+^ and Ca^2+^-dependent signal pathways are involved.

In our experiments on the crayfish stretch receptor model, we observed a significant Ca^2+^ level increase in the glial envelope of the soma and proximal axon segment [30].

## 3. Calcium in Electrophysiological Response to Axotomy

Detailed studies of electrophysiological responses of crayfish neurons to AT were conducted by Kuwada as far back as 1970–1980 [31,32]. Normally passive, not firing, somata of certain unipolar efferent crayfish neurons became electrically active in the period of 36 h post-AT. These changes persisted about two weeks, and then diminished. The decline in excitability occurred regardless of regeneration, and the excitability did not recover after repeated severing of axon stump. Additionally, in somata, normally being active, electrogenicity increased as well, which was expressed in heightened amplitude and frequency of spikes and their easier induction. Nevertheless, some classes of normally passive and active neurons did not respond to AT electrophysiologically. The extensive injury of afferent neurons did not induce changes in efferent neurons of the same ganglia, and no mutual effects with AT of efferent neurons took place either. Neurons with longer axon stumps developed soma excitability slower. The incoming ionic current in AT-induced spikes of some neurons was carried mainly by Na^+^ ions.

AT did not affect Ca^2+^ spikes, the dependence of membrane potential on extracellular calcium concentration (according to the Nernst function), and its independence on extracellular sodium. In axonal spikes, the inward current was carried mainly by sodium ions both before and after axotomy. The overshoot of axonal spikes did not change dramatically between the period of presence and absence of somatic spikes. Temperature increase accelerated both the beginning and ending of AT-induced soma spiking. AT of *Procambarus clarkia* crayfish’s anal motor neuron induces long-term spiking locally in the transection site [33]. This spiking, as well in in Kuwada experiment, is responsible for voltage-gated Na^+^ conductivity, but not Ca^2+^ conductivity. In our experiments on crayfish stretch receptor neurons, the firing frequency and the amplitude of action potentials did not change during first minute after the axon transection [19].

In cultures of mammal (murine) cortical neurons, an acute electric response to AT was observed [34]. Sensory neurons of spinal ganglia responded to AT with excitability increase [35], which is linked to AT-induced negative regulation of voltage-gated potassium channels Kv9.1 [36]. Such a difference can be associated with peripheral or central identity of neurons. However, it is quite possible that the glial envelope highly affects the character of spiking and electrophysiological response to injury, making the functional activity of neurons more stable. The buffer, which the glia created between the plasma membrane of neurons and the extracellular medium, lowers the involvement of channels, involved in the action of potential generation, in neuronal response to axon injury.

The increase in neurons excitability and spike bursts after AT is associated with the increase in intracellular Ca^2+^ concentration [35]. However, in the experiments of Hogan [37], the inward current of Ca^2+^ ions into cytosol through plasmalemma also decreased after axotomy, and its artificial recovery removes the hyperexcitability. This makes it possible to suggest that the AT-induced increase in Ca^2+^ concentration occurs not because of Ca^2+^ influx through the cell membrane, but via other pathways.

The level of intracellular Ca^2+^ mediates changes in neuronal electric activity in response to AT [35]. In [38], there was no difference in resting [Ca^2+^]_i_ between neurons, axotomized 7–10 days before, and controls. However, the elevation of [Ca^2+^]_i_, caused by orthodromic and antidromic stimulation, and the recovery after the stimulation train, were significantly lower in axotomized neurons, than in controls.

Perineuronal glial cells are a specific cell type for the peripheral nervous system and play a critical role in the conditions of peripheral nerve damage. These cells are located in optimal fashion so that, to affect the functions of sensory neurons, for example, they envelope sensory neurons in the structure of dorsal root ganglia. The barrier they create, usually one-to-three layers, slows down the diffusion of many molecules, especially large ones, which gives a certain control of perineuronal media and helps to maintain in it the required homeostasis for the action potential propagation in the sensory neurons [39].

In response to peripheral nerve damage, a number of interconnected processes occurs in neuron and satellite glia. This includes the coupling of satellite glial cells, increase in ectopic spontaneous neuronal spiking and abrupt changing in electrical characteristics of neurons and glia (membrane depolarization and decrease in membrane resistance and threshold values of membrane current for the action potential generation) [40], glial proliferation, and increase in glia–glial interaction via gap junctions [27,41]. This creates favorable conditions for the propagation of Ca^2+^ waves in the glial envelope as a result of mechanical damage or a signal from an axon [42].

Upon the disturbance of homeostasis, as in the case of peripheral nerve injury, satellite glial cells become active, demonstrating a high expression of GFAP (glial fibrillary acidic protein), and proliferate. Damage to peripheral nerves also induces an increase in the expression of the gap junction subunit connexin 43 (Cx 43) in satellite glial cells of dorsal root ganglia [27,42]. The resulting increased gap junction connectivity of satellite glial cells, surrounding individual neurons and their neighbors, performs many functions. This mechanism can also be beneficial for the neuron, providing the distribution of metabolites. The increased junction of satellite glial cells in sensory ganglia is apparently involved in various neuropathic pain conditions and can be connected with changes in the buffering of ion currents, including K^+^ ones, between these cells. Additionally, the decrease in the expression of inward rectifying K^+^ channel Kir4.1 in satellite glial cells, associated with sensory nerve AT, also affects this pain condition. Thus, changes in the levels of expression of Cx43 and Kir4.1 can indirectly affect threshold values of action potential via changing the ability of satellite glial cells to effectively buffer the extracellular perineural medium [27]. Voltage-gated Na^+^, K^+^, Cl^−^, and Ca^2+^ channels function in Schwann cells and astrocytes [39].

## 4. Calcium in Ultrastructural Response of Neurons and Glia to Axotomy

At the ultrastructural level, after AT, glial and/or vesicular seal and growth cone for regeneration can be formed. The glial seal formation on the end of severed axon appears to be typical for crustaceans. The intrusion of glial cell was more significant in areas near to the axotomy site and increased with time. Glia, absorbed in the stumps, demonstrates hypertrophy and changes in nucleus morphology. At the same time, despite serious changes in morphology, these stumps still preserved the ability to conduct action potentials and to release neurotransmitters in their synapses [43].

In CNS axon stretching, an acute elevation of intracellular Ca^2+^ occurs mainly due to its release from intracellular storages, then the more gradual and continuous dysregulation of intracellular Ca^2+^ metabolism takes place. There is a suggestion that physical impacts can induce “mechanoporation”, an opening of axolemma, causing intracellular Ca^2+^ currents. The focal elevation of Ca^2+^ in axons is linked to the formation of axonal spherical swellings. The formation of these spheroids can be prevented by blocking Na^+^–Ca^2+^ exchangers NCX-1, voltage-gated N- and L-type Ca^2+^ channels, thus probably preventing reaching the Ca^2+^ threshold level for axolemma. It is still questionable if the prevention of such processes beneficial, but, apparently, the non-controlled elevation of intracellular Ca^2+^ level can induce secondary AT. The possible mechanism of this includes active cytoskeleton degradation, mediated by Ca^2+^-dependent cleaving enzymes, activated by Ca^2+^ elevation. Furthermore, Ca^2+^ uptake by mitochondria can induce energetic dysfunction, forming reactive oxygen species and resulting in oxidative injury [1]. Cyclosporin A, an inhibitor of calcineurin and MPTP, decreases secondary axotomy and cytoskeleton degradation after stretch damage to axons [44].

The influx of Ca^2+^ in major or minor lesions urges different glial or axonal membrane vesicle structures to accumulate in the lesion site and interact, repairing the integrity of axolemma through Ca^2+^-induced fusions [45]. The sealing of axon lumen requires elevation in the concentration of intracellular Ca^2+^ and is most likely mediated by membrane fusion proteins. Ca^2+^ also regulates the interaction between synaptotagmin and syntaxin, which are believed to mediate the fusion of synaptic vesicles with axolemma, providing an opportunity for neurotransmitter release in synapses. To determine if the synaptic proteins are involved in axon sealing after the damage, the researchers administrated antibodies into giant squid and crayfish axons, which inhibited either binding Ca^2+^ ions with Syt or the Ca^2+^-dependent interaction of syntaxin with Ca^2+^-binding domain of synaptotagmin. Axons, treated with antibodies for synaptotagmin and syntaxin, were not sealed after AT, which indicates that these proteins play a significant role in repair of neuronal membrane after AT [46,47]. Calpain, a Ca^2+^-dependent protease, promotes sealing of transected axons in squid; in crayfish, calpain inhibitors prevent the sealing [48,49]. It is suggested that calpain modulates the extension of glial lamellipodia [50] and participates in ultrastructure rearrangement in the transected axonal segment, leading to the formation of a growth cone [51,52]. Ca^2+^ transient in mechanical damage also induces growth cone formation [53].

In a number of cases, axon injury leads to the proliferation of surrounding glia, expressed both in a direct increase in glial extension and an increase in the number of glial cells via their division. In experiments with giant nerve fibers of *Periplaneta americana*, the proliferation of perineural glial cells began soon after axotomy [54].

Axotomy directly damages glia, axon cytoskeleton and mitochondria, located close to the axon transection site [19].

Bcl-2 supports axon regeneration via CREB and ERK pathways, promoting Ca^2+^ regulation by the endoplasmic reticulum [55].

Glia also can directly participate in the sealing of lesion site. In some invertebrates, glia can enter the lumen of a transected axon up to its complete filling [43]. There exists a mechanism of vesicular transport of substances from glia to neuron [56,57,58,59,60,61], but it is still unknown whether the opposite process exists.

The interaction of perineuronal glia with Schwann cells plays an important role in the development and health of the PNS. Perineuronal glia form “bridges” after AT of motor nerves for the following regeneration and aim Schwann cell migration for the formation of the possibility of axon regeneration towards its target [62].

**Figure 1:**
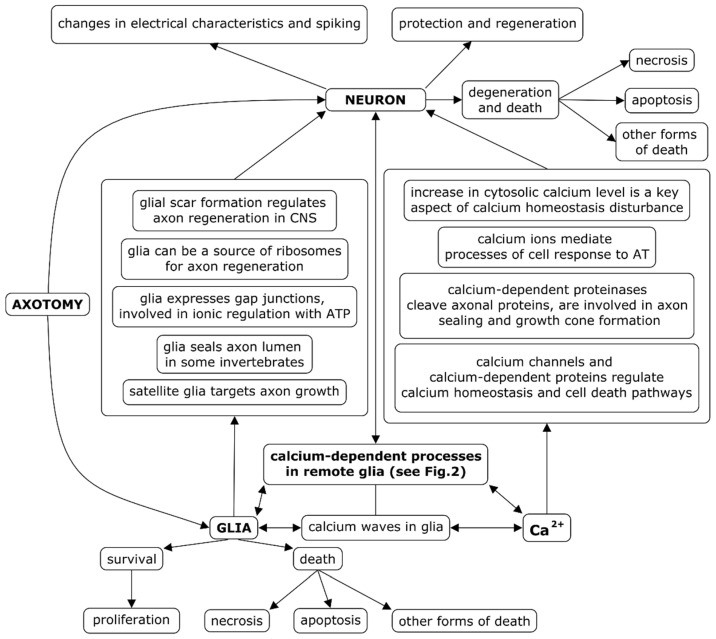
Interactions of neuron, glia, and Ca^2+^ ions in the response of neurons and glia to axotomy. Axotomy-induced processes in remote glial cells and their connection with the fate of the neuron (survival and death), as well as the role of Ca^2+^ ions in them, are relatively poorly studied.

CNS injuries often result in the formation of glial scar, which prevents axon regeneration [63,64]. Glia inhibitors can promote axon regeneration [65]. Moreover, there are studies suggesting that reactive glial scar, activated via STAT3-dependent transcription, can assist axon regrowth [15]. Furthermore, the injured axon can import ribosomes from neighboring glial cells [66]. Neurotrophic factors, modulating glia, can promote neuron survival after AT, for example, protecting them from apoptosis [67]. The facts mentioned above are summarized in Figure 1.

There is also a hypothetic possibility that entire mitochondria can be transferred between neurons and glia for mutual support and mitochondrial recirculation [68].

The mobile microglia, unlike the rest glia (macroglia) in vertebrates, develops not from the ectoderm, but from the mesoderm, and migrates to the CNS in the course of embryogenesis, henceforth implementing immune functions there [69]. Therefore, microglial responses to AT are of a different nature than AT-induced responses of perineural glia (olygodendrocytes and astrocytes in the CNS, Schwann cells in the PNS), so we do not consider them in this review.

## 5. Calcium Pathway in Death, Survival, and Regeneration of Neurons and Glia after Axotomy

Ca^2+^ is a central link of many intra- and extracellular processes in neurons and glial cells, including intercellular communication, response to trauma and post-traumatic recovery, or cell death. The disruption of Ca^2+^ homeostasis is one of the cell death factors [10,11]. The elevation of cytosolic calcium concentration from 10^−4^–10^−3^ M can trigger the cell death scenario, including necrosis and apoptosis [12].

The increase in Ca^2+^ concentration activates a number of molecular mechanisms: calpain hyperactivation and cleavage of Na^+^ channels and other axon proteins, histone deacetylation, retrograde signalization via JUN-kinase, and importin pathways [8]. The elevation of cytosolic Ca^2+^ levels causes the release of Ca^2+^ and other high-and low-molecular components from the mitochondria through MPTP, which leads to the necrosis and apoptosis of nerve cells [70]. AT can result in the depletion of calcium storages [10,71]. Changes in intracellular Ca^2+^ storages can act in concert with death signals, which initially do not require Ca^2+^, promoting the utilization of cellular components and death via apoptosis or necrosis. In addition, Ca^2+^-activated enzymes can cleave proteins, phospholipids, and DNA, inducing cell death and tissue damage.

Ca^2+^ regulates all stages of apoptosis. Excessive levels of cytosolic Ca^2+^ levels promote apoptosis via different pathways, for example, it causes Ca^2+^ entry to mitochondria and promotes the opening of MPTP, triggering the mitochondria-dependent apoptotic pathway. Calpains are Ca^2+^-dependent cysteine proteases which mediate the cleavage of some BCL-2 family apoptosis-regulating proteins BID, BCL-2, and BCL-XL. They are also responsible for the permeabilization of the outer mitochondrial membrane and the release of cytochrome c and AIF. Yet another mechanism is the activation of Ca^2+^-regulated phosphatase calcineurin. It dephosphorylates the BCL-2-associated cell death agonist (BAD), a proapoptotic member of BCL-2 family, thus increasing heterodimerization of BAD and BCL-2-XL and promoting apoptosis.

In the brain, Ca^2+^ plays a fundamental role in synapse activity control and memory formation, a process resulting in the activation of specific Ca^2+^-dependent pathways of signal transduction, and involves key protein effectors, such as calmodulin kinases, MAPK/ERK kinases, and CREB [11].

The increase in cytosolic Ca^2+^ levels promotes axon regeneration after laser transection. The in vivo application of MTPT inhibitors bongkrekic acid and cyclosporin A prevented both cytochrome c release and the following activation of kaspase-3, and decreased motor neuron apoptosis. The application of an MCU inhibitor, ruthenium amino complex RU360, made similar effects [72].

Moreover, ER stress induction, because of changes in ER Ca^2+^ homeostasis, may induce apoptosis via the caspase-12 pathway, not associated with mitochondria. Additionally, Ca^2+^-dependent splitting and deactivation of caspase-12 by calpain is allegedly a base of apoptosis caused by ischemia and glucose deprivation [73]. The increase in cytosolic Ca^2+^ is also associated with the activation of some DNA-degrading endonucleases [74].

Ca^2+^ is involved in the work of cytoskeleton-related factors; for example, caltubin, a mollusk protein, interacting with tubulin, promotes axon growth and decreases axon degeneration in rodents [75]. A number of Ca^2+^-dependent proteins are involved into responses of neurons and glia to axonal injury: calpains, calmodulin, calmodulin kinases, protein kinase C, among others.

AT promoted calmodulin expression in the crayfish ventral nerve cord [76]. The expression of calmodulin kinase II (CaMKII) beta decreases during axon regrowth, while CaMKII alpha, apparently, supports axon regrowth [77]. A post-traumatic CaMKK–CaMK1a signal pathway is induced in somatosensory neurons of mice in peripheral nerve injuries. The induction of CaMK1 is typical for neurons of dorsal root ganglia in response to peripheral neurotrauma and is a potential target for therapeutic intervention to improve the regeneration of peripheral nerves [78].

The family of protein kinase C (PKC) is a group of important signal molecules, involved in the prevention of neurodegeneration after injuries of nervous system. AT increases the level of PKC II in axotomized neurons [79]. The inhibition of protein kinase C in AT protects Purkinje cells from death, but does not affects axon regeneration [80]. Neurotrauma induces the expression of GDNF in Schwann cells via purinergic signaling and the PKC–PKD pathway [81]. The upregulation of calbindin expression can promote the survival of damaged motor neurons [82]. In cortical neurons, when PKC is inhibited by staurosporin or PKC41, the activation of calpain leads to import of extracellular Ca^2+^ through the hyperpolarization-activated plasma membrane channel HNC2. The downregulation of this channel blocks the entrance of Ca^2+^ into the cell, as well as AIF-regulated pathway and apoptosis [74].

Calpain II can translocate to the nuclei by itself or regulate apoptosis, interacting with Bcl-2 family proteins. Calpain activation can cause permeabilization of lysosomal membranes, which results in release of toxic cathepsins into cytosol. After AT, activated calpain also cleaves and inactivates Na^+^–Ca^2+^ exchanger in neurons, causing Ca^2+^ overload and necrosis, which can be prevented via calpain inhibition or expression of the exchanger, not cleavable by calpain [8]. In neurons, calpain can be inhibited by a natural inhibitor, calpastatin, which prevents their death from excitotoxicity [83].

The growth cone formation and regeneration in various types of axons is regulated by Ca^2+^, cAMP, and ERK. Regeneration in vitro also requires Ca^2+^, which apparently acts through protein kinases, such as ERK or PKA [84]. Ca^2+^ and cAMP promote axon regeneration in *C. elegans*, engaging DLK-1 kinase [85]. Bcl-2 supports axon regrowth via the increase of intracellular Ca^2+^ signaling and the activation of CREB and Erk proteins, promoting a regenerative response and neurogenesis; the expression of Bcl-2 decreases the intake and storage of Ca^2+^ in ER and thus leads to a stronger intracellular Ca^2+^ response, induced by Ca^2+^ influx or axotomy in Bcl-2-expressing neurons, than in controls [55].

S100B, a Ca^2+^-binding protein, is overexpressed in acute neural injury and its inhibition reduces the detrimental consequences of the trauma, but, in trophic concentrations, it can promote neuro- and synaptogenesis in the CNS [86].

Neurotrophic factors play a significant role in protection of neurons in case of axotomy. As far back as 1995 [87], several neurotrophic factors, protecting motor neurons, were mentioned. Ciliary neurotrophic factor (CNTF), brain-derived neurotrophic factor (BDNF), neurotrophin-4/5, and insulin-like growth factor 1 promoted the survival and regeneration of axotomized neurons on different models [67,87,88,89,90,91,92,93,94,95,96,97,98]. CNTF is an early axotomy-induced retrograde signal [99]. Post-traumatic GABA-mediated increase intracellular Ca^2+^ concentration is required for the induction of BDNF-dependent survival of adult CNS neurons [100]. The neuronal Ca^2+^ sensor 1 (NCS-1) is a survival factor for damaged neurons, mediating GDNF survival signal via the PI3K-Akt pathway [101].

It is shown that astrocytes increase during an hour the level of connexin-43, a gap junction protein, and, within a day, increase the level of multifunctional GFAP protein [42]. Similar processes occur in Schwann cells with possible modulation by inflammatory cytokines [102]. PKC and PKA phosphorylate GFAP at serine and threonine residues [103], which indicate the important role of GFAP in intercellular communication.

## 6. Cell Death Scenarios in Neurons and Glia after Axotomy

AT induces a complex cascade of metabolic and signal processes, aimed at counteraction to further lesion and compensation of damage inflicted, or the implementation of programmed cell death, apoptosis of autophagy [104]. In less favorable cases, necrosis occurs. The term “paraptosis” is suggested by some authors for a type of cell death without the morphological signs of apoptosis [105].

The important role of Ca^2+^ in cell death regulation has been known for a long time. Necrotic cell death has, for a long time, been associated with intracellular Ca^2+^ overload, leading to membrane permeabilization and the functional collapse of mitochondria. In further specification of the signal pathways of apoptosis, it was found that Ca^2+^/calpain is critically involved in the work of apoptosis induction factor (AIF). Later, it was shown that Ca^2+^ plays an important regulatory role in other types of cell death, in particular, autophagy and anoikis [74].

The overexpression of anti-apoptotic protein Bcl-2 [106] and deletion of proapoptotic protein Bax [107] prevent AT-induced cell death. Bcl-2 protects AT-induced apoptotic death in motor neurons [108]. The in vivo application of MPTP inhibitors blocks apoptosis triggering in axotomized facial neurons of new-born mice [72]. As a consequence of secondary pathophysiological mechanisms, caused by spinal injury, olygodendrocytes die, being subjected to apoptotic “waves” [109,110]. In neurons, L-glutamate, an inductor of Ca^2+^ inflow and calcineurin activation, induced the translocation of Bad to mitochondria and apoptosis, which can be inhibited by the co-expression of a mutant inhibitory form of calcineurin and its pharmacological inhibitors [74].

Injured axons are a place of reactive oxygen production, energy supply disruption, and MPTP formation [1]. The inhibition of MPTP with cyclosporin A protects neurons after axonal damage [111], and also protects astrocytes from necrosis [112].

Some types of CNS injuries involve changes in mitochondrial potential and metabolism both in neurons and astrocytes linked to MPTP opening [68].

**Figure 2:**
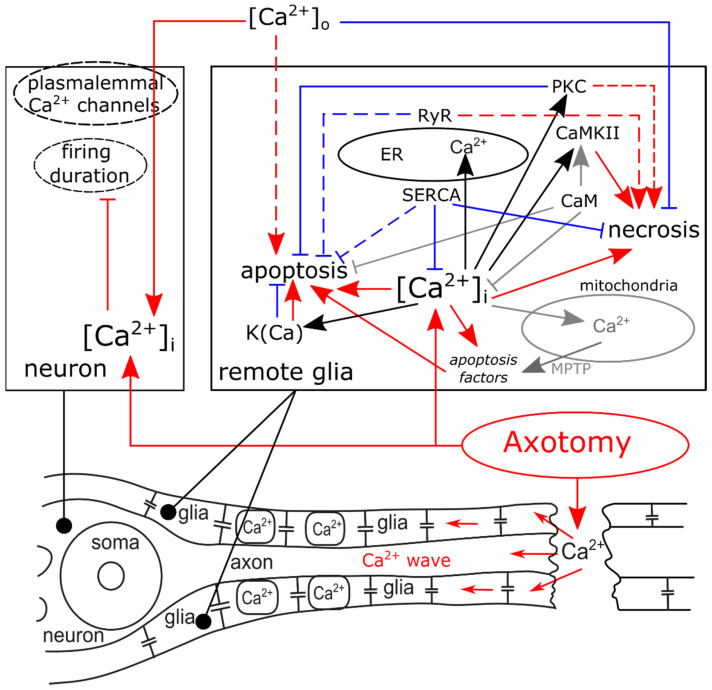
Involvement of Ca^2+^ ions, Ca^2+^ channels, and Ca^2+^-dependent proteins in death or survival of remote glial cells (RGC) in crayfish stretch receptors after axotomy (AT), based on our experimental data. AT induces Ca^2+^ influx into axolemma and the elevation of Ca^2+^ levels in cytosol of neuron and glia ([Ca^2+^]_i_), promoting the cessation of neuron firing and glial death. The work of SERCA, pumping Ca^2+^ from cytosol, protects remote glial cell from necrosis and apoptosis. Protein kinase C (PKC) and calmodulin kinase II (CaMKII) are involved in the necrosis of RGC, but PKC decreases their apoptosis, as well as Ca^2+^-dependent potassium channels K(Ca). Extracellular Ca^2+^ ([Ca^2+^]_o_), promotes apoptosis, but decreases the necrosis of RGC. Sharp arrowheads: increase effects. Blunt arrowheads: decrease effects. Dashed lines: the effect takes place only after AT. Red: detrimental effects. Blue: protective effects.

In crayfish stretch receptors, AT not only decreased firing duration (the functional “lifetime”) of mechanoreceptor neurons [19], but also promoted necrosis and apoptosis of remote glial cells (RGC) [30,113]. Eight hours of incubation of a sample in saline are sufficient for the appearance of morphological signs (fragmented nuclei) of spontaneous or induced glial apoptosis. The apoptosis did not increase in the following hour of incubation in intact samples (so it is a spontaneous apoptosis), but continued to grow in axotomized cells [113], thus suggesting that AT is a long-lasting damaging factor, although the lumen was instantly sealed mechanically after the transection [19]. Our further analysis showed that AT makes remote glia susceptible to apoptosis promoted by increased extracellular Ca^2+^ and impaired the functions of ryanodine receptors and ER Ca^2+^ ATPase (SERCA). As for necrosis, AT-specific pro-necrotic functions of ryanodine receptors and protein kinase C were detected [30]. The summary is given in Figure 2.

Despite the fact that autophagy can be both protective [83,104] in axotomized neurons and also is linked to Ca^2+^-related homeostasis disruptions, such as ER stress [114,115], the topic of how Ca^2+^ is involved in AT-induced autophagy remains understudied. Autolysis (lysosomal cell death) can occur if calpain or other calpain-dependent enzymes destroy lysosomal membrane, releasing cathepsins (DNAses and lipases) and reactive oxygen species, which increases the acidity of intracellular medium [83]. There are still no direct data about AT-induced autolysis. Sarmoptosis is a SARM1-dependent neuron death, usually induced by AT. Mitochondrial dysfunction, which includes Ca^2+^ influx, induces sarmoptosis in sensory neurons [83,116].

## 7. Future Prospects and Targets

As we said above, AT promotes the activity and expression of PMCA. At present, direct PMCA inhibitors are not easily accessible, so indirect methods of inhibiting this pump are used, for example, the increase in saline pH against SERCA inhibition [24].

IP3 receptors (IP3R) are, together with RyR, a way of Ca^2+^ release from ER. Calcium signaling in astroglia is based on combined work of IP3R store-operated Ca^2+^ channels (SOCC) in plasma membrane, belonging to the Orai family and acting together with STIM 1 and 2 molecules, which transmit to them the ER depletion signal [117]. As in the case of PMCA, these channels can contribute to axotomy-induced increase in the Ca^2+^ concentration in remote glia.

Mitochondrial Ca^2+^ uniporter (MCU), through which Ca^2+^ enters the mitochondria, and mitochondrial Na^+^–Ca^2+^ exchanger (NCX), releasing Ca^2+^ from mitochondria, regulate Ca^2+^ movement between mitochondria and cytosol, thus being involved in Ca^2+^ homeostasis and the survival of the cell. For MCU inhibition, ruthenium complexes Ru360 and Ru265 are used [118]. There are also selective inhibitors for mitochondrial NCX (CGP-37157) [119].

There is a question about the nature of cause-and-effect processes, linking axon damage and the elevation of Ca^2+^ concentration in RGC. To study a possible mechanism of AT-induced retrograde propagating of Ca^2+^ wave along glial envelope, it seems reasonable to apply gap junction inhibitors, such as arachidonic acid.

Thus, the analyses of the involvement of PMCA, mitochondrial Ca^2+^ uniporter and NCX, IP3R with SOCC, and differentiated Ca^2+^-dependent potassium channels are a prospective direction in the research of Ca^2+^ mechanisms, regulating AT-induced death and the survival of RGC together with neurons. The wide spectrum of Ca^2+^-activated proteins also needs to be comprehensively studied for potential therapeutic targets.

## 8. Conclusions

Traumatic brain injury is one of main causes of death and disability in young and middle age, and spinal brain injury is one main causes of disability, limiting mobility in people of all ages. Peripheral nerve injuries remain a growing social-economic burden, mainly affecting the young working population. The existing methods of clinical treatment, aimed to prevent the death and degeneration of nerve cells in the first hour after a trauma, are, in general, insufficient, leaving a significant part of motor or sensory functions lost.

One of the recovery conditions after such injures is the preservation of viability of damaged neurons and glia, which, in turn, depends on the number of factors, including Ca^2+^ homeostasis and neuroglial interaction. To date, biomedical science has not created sufficiently effective agents and methods of treatment, aimed at both of these factors and considering their connection.

The death and survival of glia have substantial significance in the recovery after neurotrauma, in which, on the one hand, glia plays protective role, and on the other hand, glia should not hinder the regeneration processes. There should be some type of balance between survival (proliferation) and manageable cell death (apoptosis, controlled necrosis, and autophagy). Apparently, some ways of altering Ca^2+^ concentrations increase or decrease both apoptosis and necrosis, and others, depending on circumstances, act more selectively, preventing or promoting a certain type of death. The important fact is that AT induces susceptibility to activation or inhibition of certain signal pathways.

The already obtained data about the involvement of Ca^2+^ regulation mechanisms in AT-induced death of RGX indicate some possible directions for the search of novel and the development of existing methods of pharmacological applications for protecting neurons and glia from the consequences of neurotrauma. Nevertheless, the interplay between neurons, satellite glial cells, and Ca^2+^-dependent mechanisms in response to AT, despite its critical importance, especially for clinics, demands further research. Many questions remain unanswered, and many problems remain unresolved. We hope that this review will help to conceptualize the problem and, with the help of our recent data, to build a roadmap and perform new productive studies for more integrative practical understanding of what is happening with neurons and their environment after axotomy and what can be done to protect them and facilitate the subsequent regeneration and functional recovery.
